# Supplementary figures and images for: Dual-mode recognition of tRNAPro isoacceptors by Toxoplasma gondii Prolyl-tRNA synthetase
Source: EMBO Rep. 2025 Apr 28;26(11):2931–44. doi: 10.1038/s44319-025-00457-x (PMC12152171; doi:10.1038/s44319-025-00457-x)

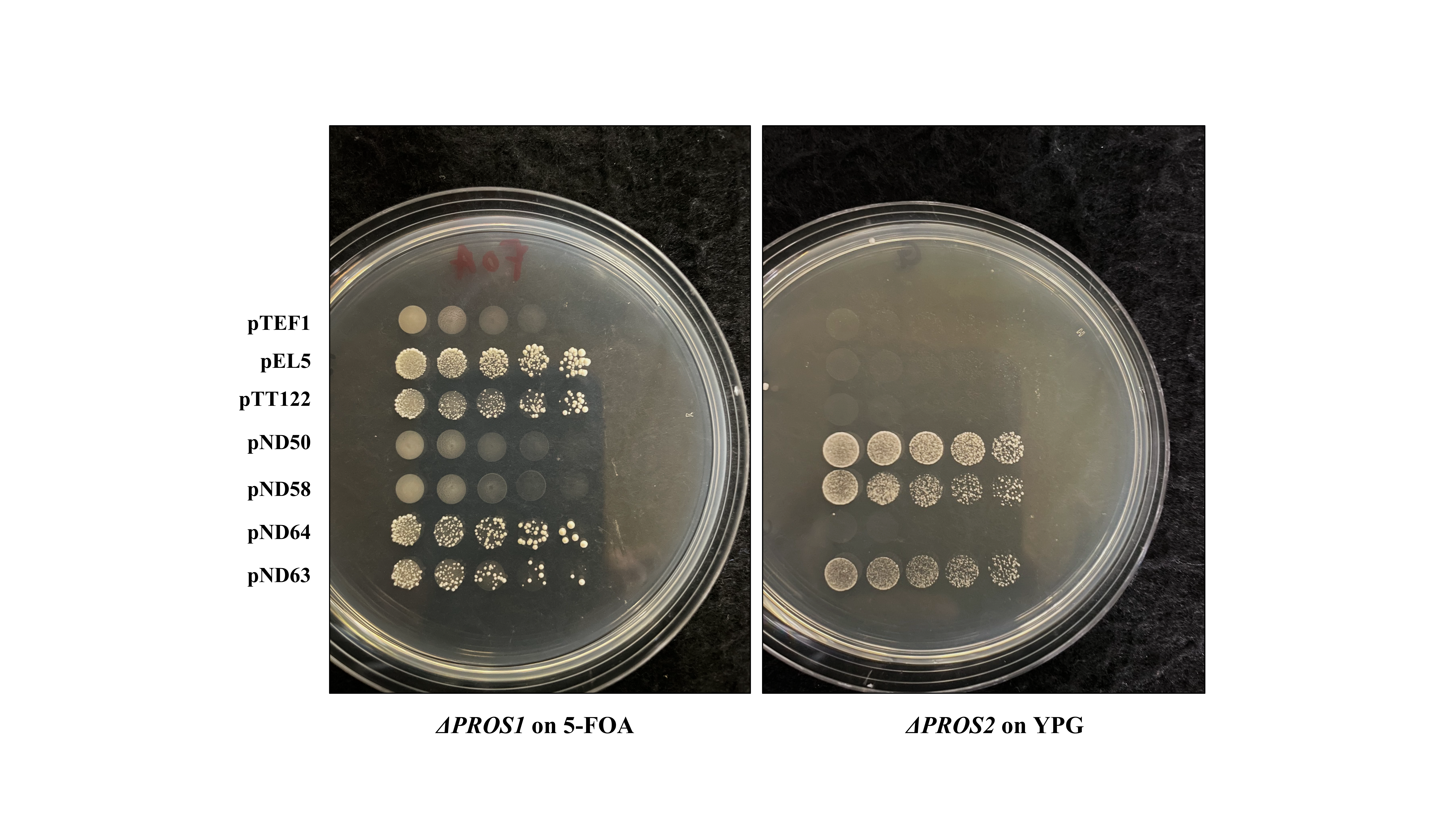

Supplement: Supplementary file 5 — Source data Fig. 3 [file 44319_2025_457_MOESM5_ESM.zip › Figure 3/3B/Whole mount of Figure 3B.tif]

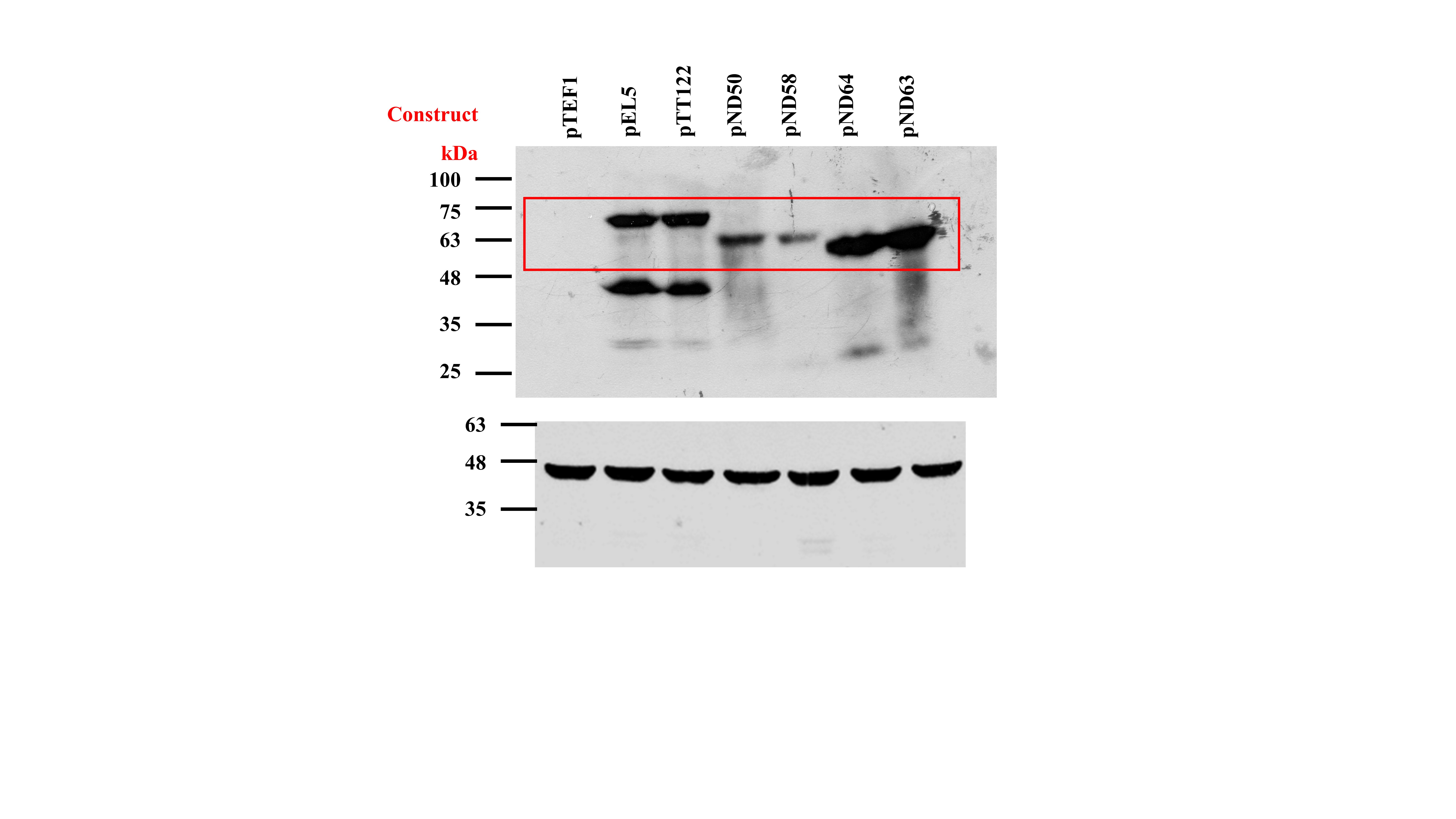

Supplement: Supplementary file 5 — Source data Fig. 3 [file 44319_2025_457_MOESM5_ESM.zip › Figure 3/3C/Blot of Figure 3C.TIF]

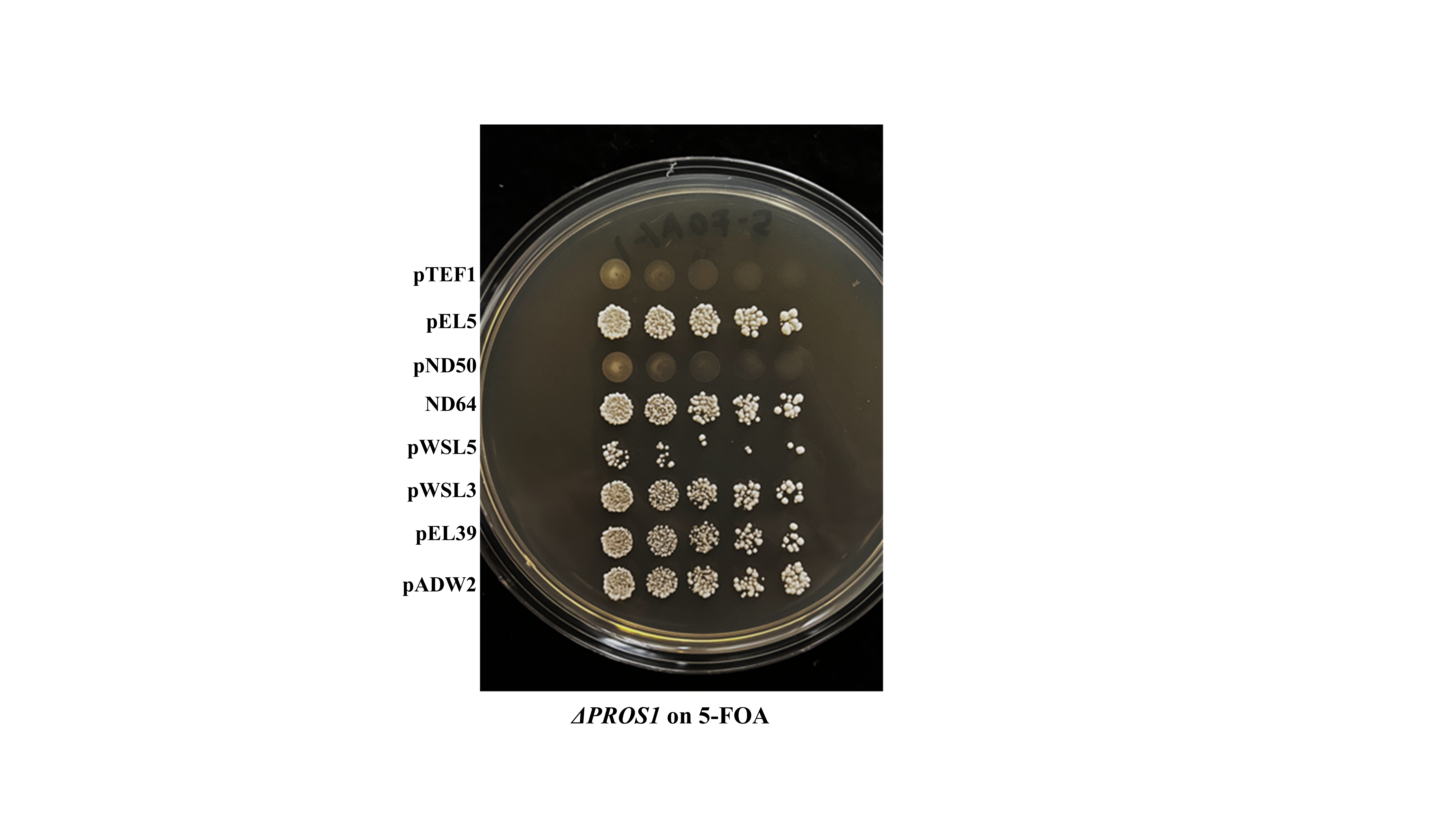

Supplement: Supplementary file 6 — Source data Fig. 4 [file 44319_2025_457_MOESM6_ESM.zip › Figure 4/4A/Whole mount of Figure 4A.tif]

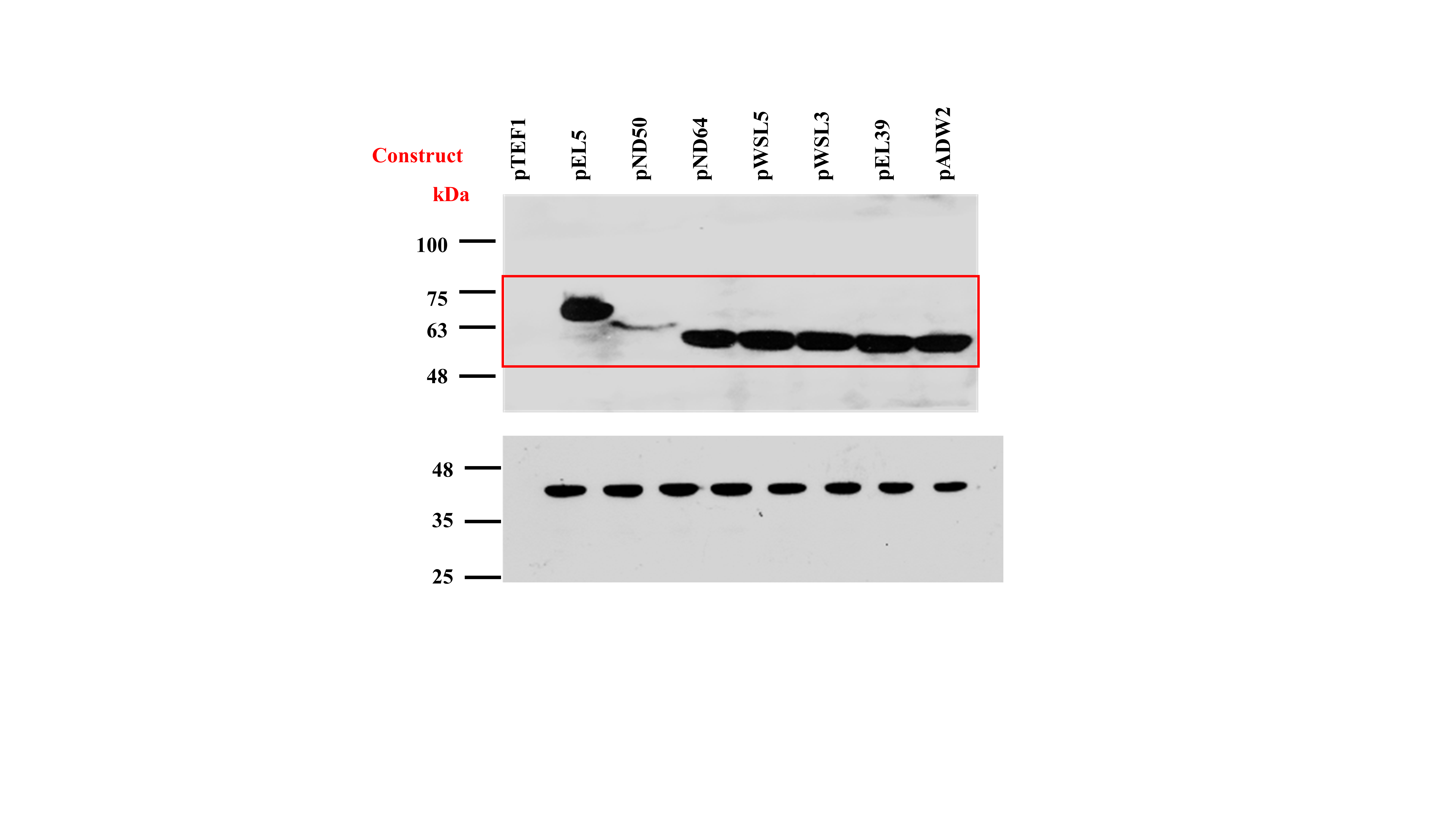

Supplement: Supplementary file 6 — Source data Fig. 4 [file 44319_2025_457_MOESM6_ESM.zip › Figure 4/4B/Blot of Figure 4B.tif]

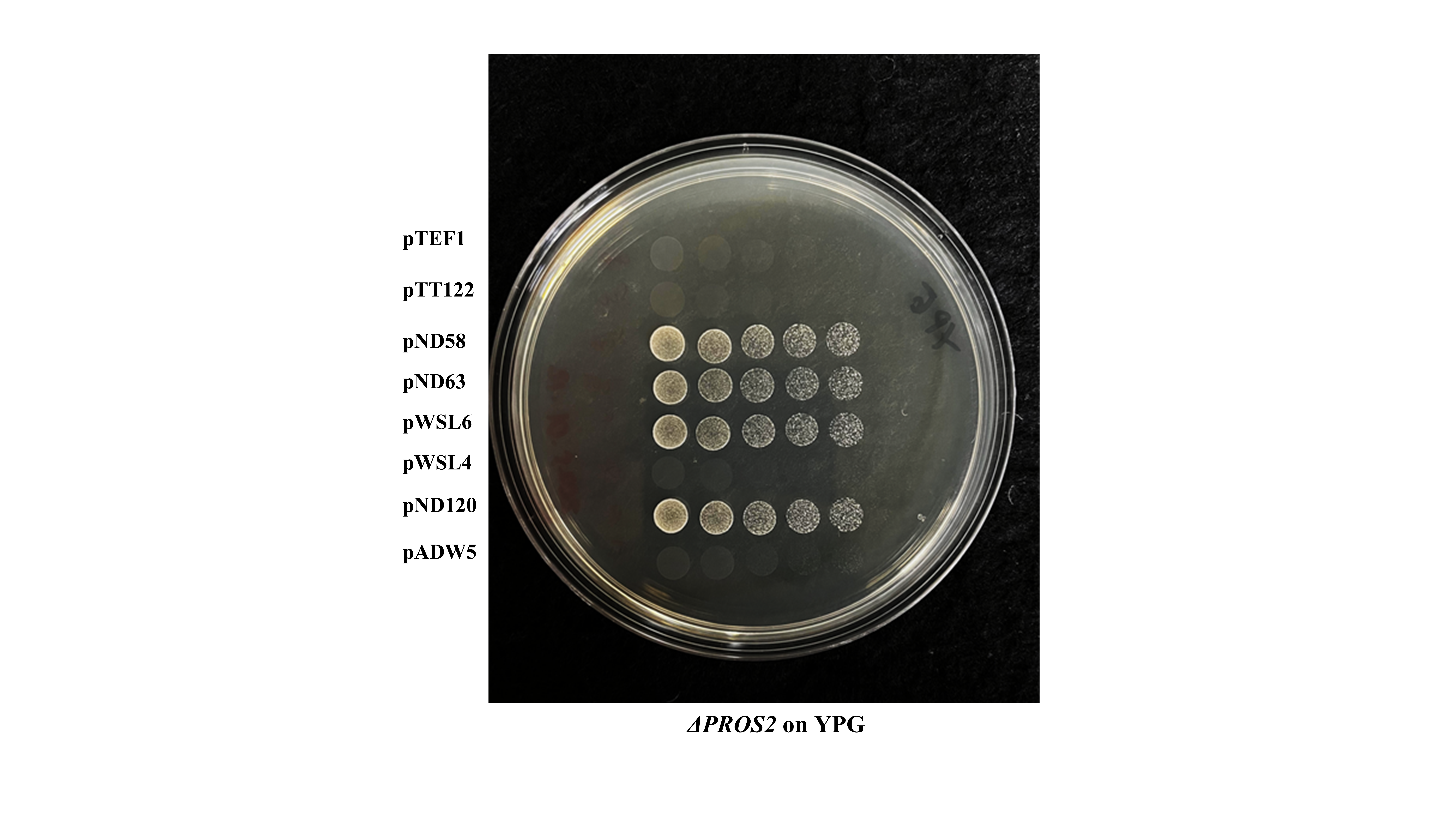

Supplement: Supplementary file 6 — Source data Fig. 4 [file 44319_2025_457_MOESM6_ESM.zip › Figure 4/4C/Whole mount of Figure 4C.tif]

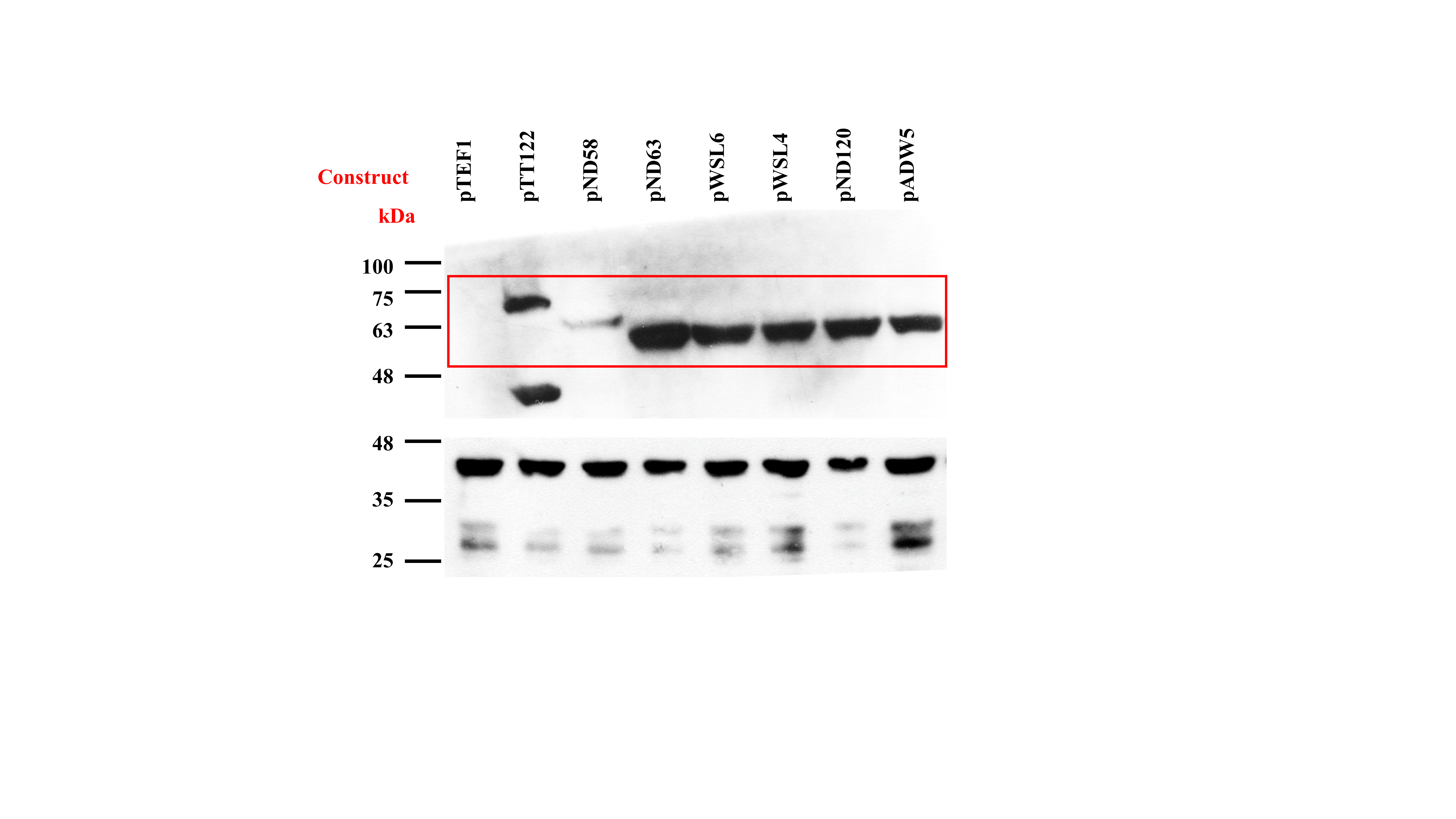

Supplement: Supplementary file 6 — Source data Fig. 4 [file 44319_2025_457_MOESM6_ESM.zip › Figure 4/4D/Blot of Figure 4D.tif]
