# Supplementary material for: Dual-mode recognition of tRNAPro isoacceptors by Toxoplasma gondii Prolyl-tRNA synthetase
Source: EMBO Rep. 2025 Apr 28;26(11):2931–44. doi: 10.1038/s44319-025-00457-x (PMC12152171; doi:10.1038/s44319-025-00457-x)
Supplement: Supplementary file 8 — Expanded View Figures [file 44319_2025_457_MOESM8_ESM.pdf]

Expanded View Figures

Figure EV1. P-type $tRNA^{Pro}$ isoacceptors from bacteria and eukaryote's organelles.

Cloverleaf structure of $tRNA^{Pro}$. $TgtRNA_a^{Pro}$, *T. gondii* apicopast-encoded apicoplast $tRNA^{Pro}$; $EctRNA^{Pro}$, *E. coli* $tRNA^{Pro}$; $BttRNA^{Pro}$, *Bacillus thuringiensis* $tRNA^{Pro}$; $EftRNA^{Pro}$, *Enterococcus faecalis* $tRNA^{Pro}$; $BstRNA^{Pro}$, *Bacillus subtilis* $tRNA^{Pro}$; $HstRNA_m^{Pro}$, *Homo sapiens* mitochondrial-encoded mitochondrial $tRNA^{Pro}$; $MmtRNA_m^{Pro}$, *Mus musculus* mitochondrial-encoded mitochondrial $tRNA^{Pro}$; $AttRNA_m^{Pro}$, *Arabidopsis thaliana* mitochondrial-encoded mitochondrial $tRNA^{Pro}$; $DmtRNA_m^{Pro}$, *Drosophila melanogaster* mitochondrial-encoded mitochondrial $tRNA^{Pro}$.

© The Author(s)

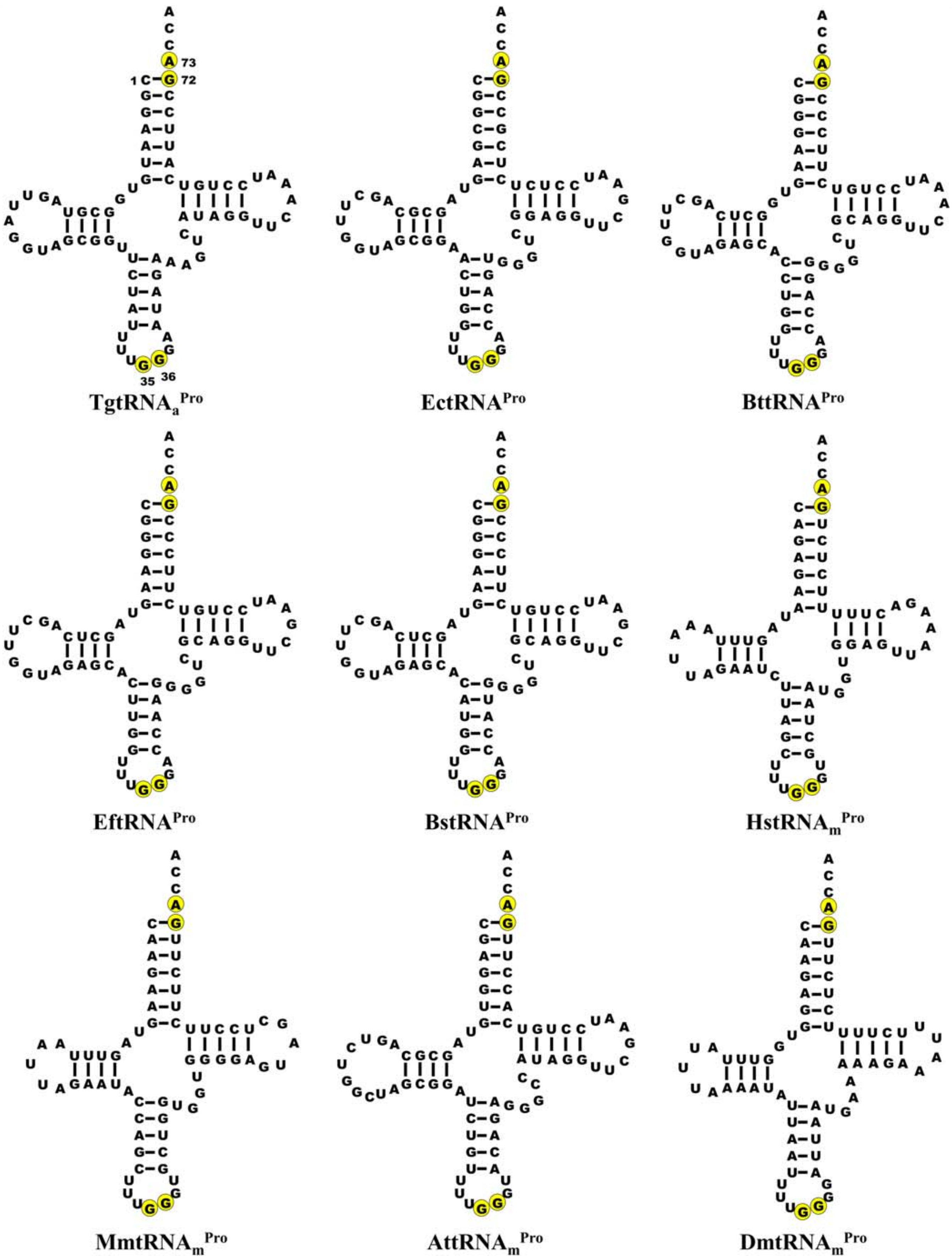
TgtRNAa Pro
EctRNA Pro
BttRNA Pro
EftRNA Pro
BstRNA Pro
HstRNAm Pro
MmtRNAm Pro
AttRNAm Pro
DmtRNAm Pro
